# Advances in the application of bone turnover markers for pediatric growth and developmental disorders: a review

**DOI:** 10.3389/fendo.2025.1615712

**Published:** 2025-09-10

**Authors:** Yuxin Wang, Honghua Hu, Yi Huang

**Affiliations:** ^1^ School of Medicine, University of Electronic Science and Technology, Chengdu, China; ^2^ Department of Laboratory Medicine and Sichuan Provincial Key Laboratory for Human Disease Gene Study, Sichuan Provincial People’s Hospital, School of Medicine, University of Electronic Science and Technology of China, Chengdu, China

**Keywords:** bone turnover markers, children and adolescents, bone metabolism, pediatric bone health, pediatric developmental disorders

## Abstract

Bone turnover markers (BTMs) are biomedical indicators used to assess the bone metabolism processes reflecting the activity of osteoblasts and osteoclasts. During childhood and adolescence, bone metabolism is highly active, leading to distinct levels and trends of BTMs compared with those of adults. BTMs correlate significantly with age, gender and environmental factors, making them valuable for evaluating bone health and developmental trajectories in pediatric populations. Due to the non-invasive characters and dynamic monitoring capabilities, BTMs are increasingly employed in research and clinical practice. Preliminary observations propose that BTMs demonstrate clinical utility in predicting fracture risk, enabling early diagnosis of osteoporosis and rickets, and monitoring therapeutic efficacy. However, Tracability of BTM measurement results and limited pediatric reference intervals remain critical challenges. Further research is needed to expand our understanding of the their mechanisms and optimize clinical applications. This article reviews the physiological and pathological states in children, discusses the current dilemmas of clinical application, and highlights the future research prospects.

## 1 Introduction

Bone is a vital structural and metabolic organ, providing mechanical support and participating in mineral homeostasis. As a dynamic tissue, bone undergoes continuous remodeling through two counterbalanced processes: osteoblast-mediated formation and osteoclast-driven resorption (1). Clinically, dual-energy X-ray absorptiometry (DXA) and quantitative computed tomography (QCT) are gold-standard techniques for assessing bone mineral density (BMD) and content due to their high accuracy and rapid results (2). However, their utility in longitudinal bone metabolism monitoring is constrained by radiation exposure risks and cost limitations. In pediatric populations, these limitations are compounded by reduced measurement precision, primarily due to motion artifacts and patient noncompliance during imaging (1, 2). BTMs metabolite or enzyme released during bone remodeling—reflect real-time osteoblast or osteoclast activity. These minimally invasive biomarkers enable dynamic monitoring of systemic bone metabolism (3, 4), contrasting with bone mineral content (BMC) and bone mineral density (BMD), which provide only a static assessment of bone mass (5).

Pediatric bone metabolism differs significantly from adults, integrating both developmental growth and remodeling processes. Studies indicate that pediatric BTMs exhibit 5- to 20-fold higher concentrations compared to adults (6, 7). As dual-purpose indicators, BTMs not only evaluate bone metabolic status but also serve as proxies for tracking growth velocity and maturation patterns. These attributes position BTMs as essential tools for early diagnosis, disease classification, and therapeutic surveillance in pediatric growth disorders (2, 3, 8).

## 2 Literature search and selection criteria

A comprehensive search was conducted by using keywords and MeSH terms to identify studies related to BTMs in children and adolescents. The keywords ‘children and adolescents’ and ‘bone turnover marker’ were employed, along with specific disorders and specific markers such as ‘osteocalcin’, ‘type I procollagen N-terminal propeptide’, ‘N-terminal cross-linked terminal peptide’, and ‘C-terminal cross-linked terminal peptide’. The search was carried out across multiple databases, including Web of Science, Google Scholar, and PubMed. Studies were included if they focused on the measurement, interpretation, reference ranges, or clinical utility of BTMs in children and adolescents (aged 0–18 years). Original research articles, systematic reviews, meta-analyses, and relevant clinical guidelines were prioritized. Animal studies, case reports, articles not in English, and studies exclusively in adults were excluded.

## 3 Characteristics of children’s bone metabolism

Bone mass accrual predominantly occurs during childhood developmental stages, with accelerated deposition observed in early childhood and adolescence periods characterized by rapid skeletal growth and critical mineralization windows (5, 9, 10). During childhood and adolescence, BTMs primarily reflect growth plate activity, while bone modeling processes remain active. Pediatric bone remodeling rates are approximately threefold higher than those in adults (11). Elevated BTM levels in children and adolescents reflect heightened remodeling activity, a physiological adaptation to mechanical loading during growth. Notably, BTMs demonstrate an inverse correlation with bone mineral density (BMD), suggesting a predominance of formation over resorption (12). Longitudinal studies indicate that approximately 90% of peak bone mass (PBM) is attained by early adulthood, reaching maximal density during Tanner stage III pubertal development, stabilizing thereafter, and declining progressively with advancing age (12, 13). Given that childhood skeletal development establishes a critical foundation for lifelong bone health, dynamic monitoring of bone metabolism and maturation patterns during this period is increasingly recognized as essential.

## 4 Bone turnover markers

**Figure 1:**
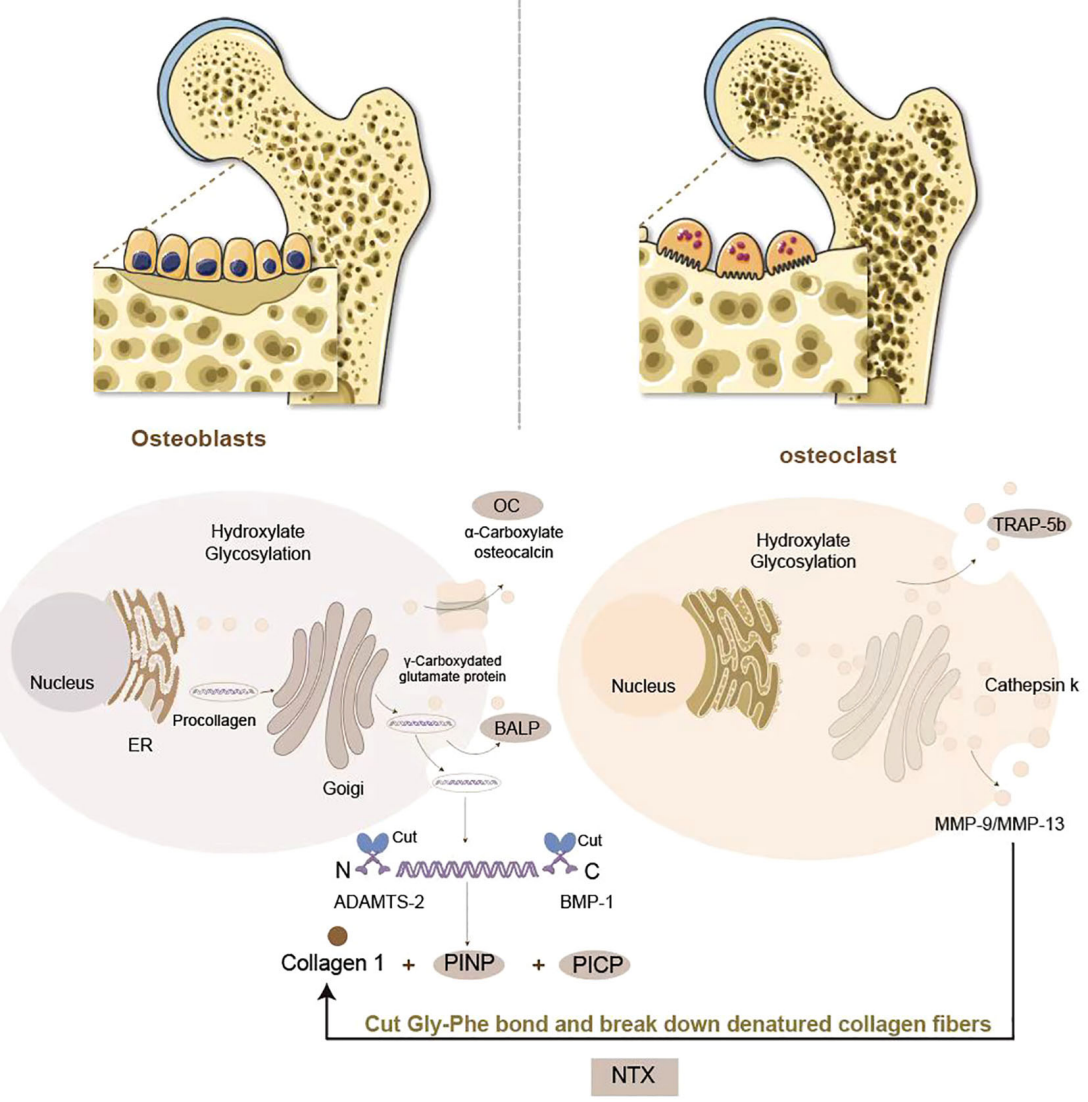
Generation of BTMs in bone formation and resorption: Osteoblasts orchestrate bone formation through type I collagen biosynthesis. PINP and PICP are produced by procollagen digestion, which serve as biomarkers of osteoblastic activity. Osteoblasts secrete BALP to facilitate hydroxyapatite deposition and OC to regulate mineralization. Osteoclasts mediate bone resorption via collagenolytic degradation. Upon attachment to the bone matrix, osteoclasts secrete tartrate-resistant acid phosphatase 5b (TRAP-5b) and create an acidic microenvironment through vacuolar H^+^-ATPases, activating cathepsin K and matrix metalloproteinases (MMP-9/MMP-13). These enzymes cleave the Gly-Phe bond in collagen’s triple helix, releasing NTX and CTX as biomarkers of osteoclastic activity.

**Figure 2:**
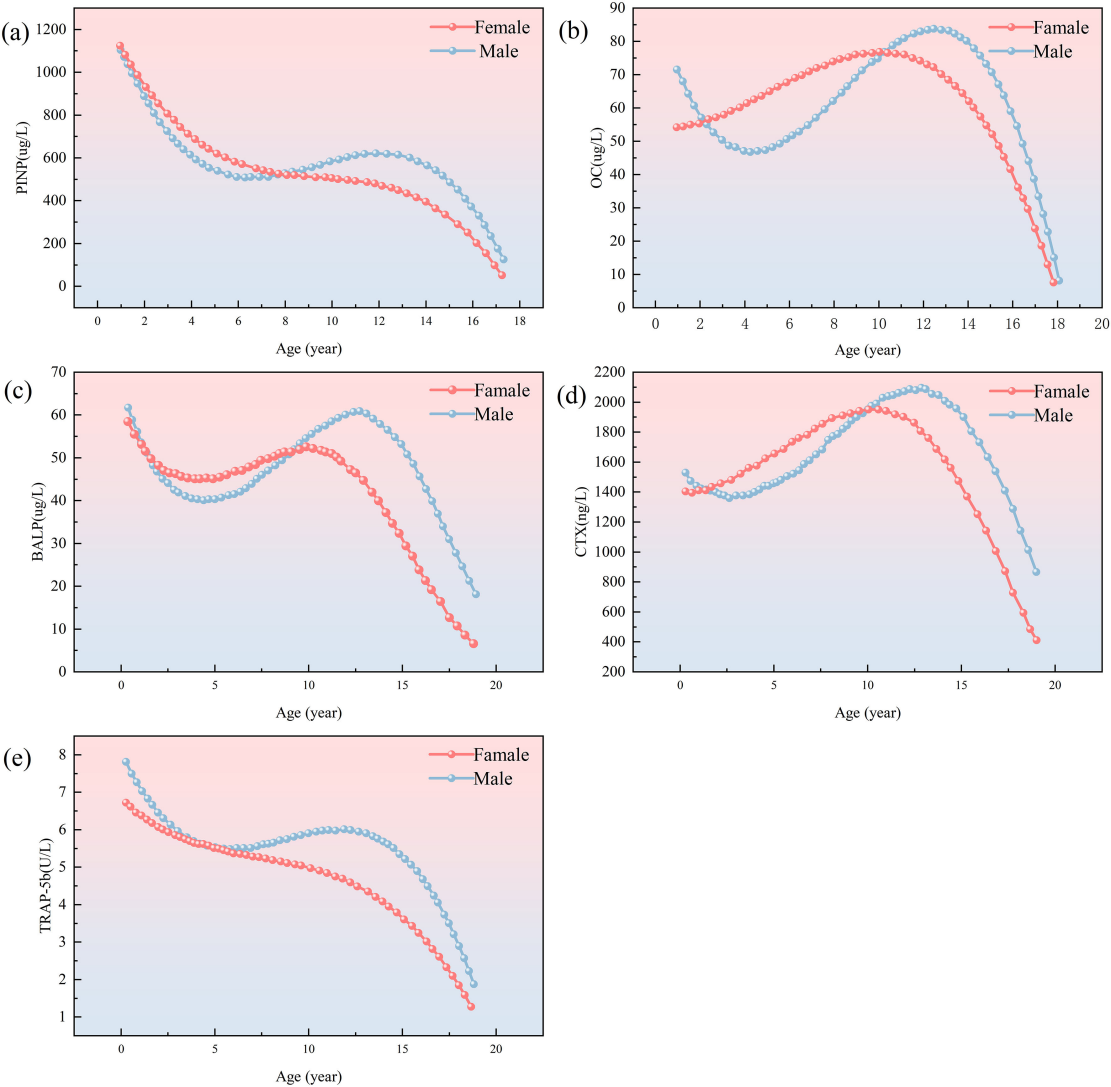
Trend charts for bone formation markers PINP, BALP, OC, CTX and TRAP-5b in male (blue dotted line) and female (red dotted line). Graph **(a)** shows PINP levels, decreasing with age for both genders. Graph **(b)** shows OCwith a peak in childhood. Graph **(c)** depicts BALP with similar trends. Graph **(d)** showsCTX levels peaking around age 15. Graph **(e)** illustrates TRACP-5b levels, decreasing asage increases. Modified and cited from Choi, J. S et al. (2019) (17), Rauchenzauner, M et al. (2007) (18).

Bone turnover markers are non-invasive biomarkers, obtained from blood or urine samples, that provide a dynamic assessment of bone metabolism (14). These biomarkers originate from the coupled activities of osteoblasts and osteoclasts. During the formative phase, osteoblasts orchestrate type I collagen biosynthesis and secretion of non-collagenous regulatory proteins, including osteocalcin (OC) and bone-specific alkaline phosphatase (BALP). Proteolytic cleavage of procollagen during extracellular matrix maturation generates quantifiable fragments such as procollagen type I N-terminal propeptide (PINP), serving as specific surrogates for osteoblast activity. In contrast, osteoclast-driven resorption involves enzymatic degradation of the collagenous matrix, liberating degradation byproducts like C-terminal telopeptide of type I collagen (CTX) and N-terminal telopeptide (NTX), which directly correlate with osteoclast functional status (15) (**Figure 1**). Historically, urinary BTMs were widely utilized due to their direct association with renal excretion of bone resorption byproducts, but due to the difficulty of collecting urine samples and the fact that BTMs values are limited by creatinine levels, serum or plasma samples are now preferred by laboratories to test for BTMs, which are easier to process and have a high degree of stability (2, 16). In 2010, the International Osteoporosis Foundation (IOF) and the International Federation of Clinical Chemistry and Laboratory Medicine (IFCC) established serum PINP and β-CTX as reference biomarkers for bone metabolism dynamics, a designation later formalized by the IFCC Bone Marker Standards Working Group to recognize PINP and β-CTX as the gold-standard biomarkers for bone formation and resorption respectively (2, 14). These designations have solidified BTMs as essential clinical tools for diagnosing metabolic bone disorders, monitoring therapeutic responses, and evaluating treatment adherence. In pediatric populations, serum levels of BTMs rise markedly during puberty, correlating strongly with growth velocity, peak bone mass accrual, and biological sex (**Figure 2**). Consequently, BTMs provide sensitive, dynamic indices for tracking remodeling processes and detecting early bone mass abnormalities (8, 19).

### 4.1 Bone formation markers

#### 4.1.1 PINP/PICP

Procollagen type I N-terminal propeptide (PINP) and C-terminal propeptide (PICP) are enzymatically cleaved fragments derived from the C-terminal domain of type I collagen during osteoblast-mediated biosynthesis. These peptides serve as specific biomarkers of bone formation, reflecting collagen I synthesis rates and osteoblast activity (3). Because PINP resistant to freeze-thaw cycles, it is easier and more economical to measure than PICP, it is more widely used in clinical practice (20, 21). The Bone Marker Criteria Working Group of the Osteoporosis Foundation and the International Federation of Clinical Chemistry and Laboratory Medicine have recommended the use of serum PINP as a reference marker for evaluating bone formation (17, 22). Significant differences in PINP between age and sex have been observed in many studies. Serum concentrations of PINP peak during infancy, decline intermittently through childhood, and diverge markedly during puberty. Adolescent males exhibit sustained PINP elevation correlating with delayed peak height velocity, whereas females demonstrate progressive declines post-menarche. These patterns mirror sex-dimorphic endocrine regulation of skeletal maturation and growth plate dynamics (23, 24).

#### 4.1.2 ALP/BALP

Bone-specific alkaline phosphatase (BALP), a tissue-specific isoform of alkaline phosphatase (ALP), is exclusively synthesized by osteoblasts and serves as a specific biomarker of osteoblast activity. While total ALP exhibits limited diagnostic specificity due to its expression in hepatobiliary and intestinal tissues, BALP demonstrates superior specificity for skeletal pathology in pediatric populations, particularly given the low incidence of hepatic comorbidities in children (25, 26). Notably, BALP quantification is minimally confounded by renal dysfunction, establishing its utility as the preferred biomarker for bone metabolism assessment in hemodialysis patients (27). During growth periods, BALP and PINP exhibit parallel trajectories, peaking during early-to-mid puberty with strong correlations to height velocity. Sexual dimorphism is evident: adolescent males display higher peak BALP and PINP concentrations occurring later than females, reflecting androgen-driven growth plate maturation (18, 28, 29).

#### 4.1.3 OC

OC (osteocalcin), a 49-amino acid γ-carboxyglutamic acid-containing protein secreted by osteoblasts, regulates calcium deposition and hydroxyapatite crystallization within the bone matrix (3). OC increases with age, peaking at 11–13 years of age in boys and 9–12 years of age in girls. Unlike BALP and PINP, the pubertal peak of OC may be lower in boys than in girls (17). Unlike PINP and BALP which reflect collagen synthesis and mineralization phases respectively, OC demonstrates dual functionality: while classically categorized as a formation marker, emerging evidence implicates OC in coupled bone turnover through paracrine modulation of osteoblast-osteoclast cross-talk (30, 31). Controversy persists regarding OC’s primary role, with some studies proposing its classification as a turnover biomarker given its homeostatic regulation of formation-resorption equilibrium independent of mineralization (17, 32). Methodologically, OC exhibits pronounced diurnal variation, with the highest concentration in the morning, necessitating standardized morning phlebotomy to ensure analytical reliability (17, 33).

### 4.2 Bone resorption markers

#### 4.2.1 NTX/CTX

N-terminal telopeptide (NTX) and C-terminal telopeptide (CTX) of type I collagen are osteoclast-derived proteolytic fragments released during collagen I degradation—the primary collagenous component of bone matrix. These telopeptides serve as specific biomarkers for quantifying bone resorption rates and metabolic turnover (34). While NTX is predominantly urinary-excreted, CTX exists in both serum and urine. Due to its ease of measurement, CTX is commonly measured in clinical settings. By specifically targeting β-isomerized aspartic acid residues within the C-terminal telopeptide region of type I collagen, β-CTX immunoassays have emerged as the gold-standard clinical methodology due to their enhanced epitope specificity and consistently superior analytical performance demonstrated in cross-laboratory validation studies (3, 34). CTX expression is relatively stable and slightly increased in childhood, peaks in early adolescence, and subsequently decreases (35, 36). serum β-CTX levels exhibit gradual prepubertal increases in both sexes, followed by sexual dimorphism during puberty. Females show stabilization post-menarche correlating with earlier growth plate closure, whereas males maintain ascending β-CTX levels until late puberty, reflecting prolonged endochondral ossification (34, 36). Studies have shown that CTX is more susceptible to diet, circadian patterns and renal function than PINP. Data suggest that CTX levels are inaccurately increased in patients with significant renal impairment and may be five times higher in hemodialysis patients. Standardized morning fasting sampling is therefore recommended to minimize pre-analytical variability (14, 37).

#### 4.2.2 TRAP-5b

Tartrate-resistant acid phosphatase (TRAP), a lysosomal enzyme secreted by osteoclasts and monocyte-macrophage lineage cells, exists as two isoforms: TRAP-5a and TRAP-5b. TRAP-5b, being osteoclast-specific, serves as a robust biomarker for quantifying osteoclast numbers and bone resorption activity (38). TRAP-5b exhibits childhood-specific patterns, with a maximum value at birth followed by an accelerated and sustained decline during puberty (18). Unlike CTX, TRAP-5b directly correlates with osteoclast population size and is less affected by renal function, so TRAP-5b can be used to measure bone resorption in patients with impaired renal function (14, 39).

## 5 Clinical applications

Bone metabolism markers are mostly used to assess bone health and bone metabolism status, and the most clinically used BTMs are PINP and CTX, which reflect bone formation and bone resorption respectively (2, 14, 40). BTMs play an important role in the assessment of bone metabolism-related disorders and the monitoring of bone metabolism modulators therapy. **Table 1** summarized clinical guidelines and consensus for BTM use in bone disorders.

**Table 1:** Clinical guidelines for BTM use in bone disorders.

Clinical guideline (Organization/Country)	Populations	Main viewpoint
Global Consensus Recommendations on Prevention and Management of Nutritional Rickets (41)	Pediatric/ adolescent	Elevated serum ALP has been used as a screening tool for rickets.
American Association of Clinical Endocrinologists and American College of Endocrinology Clinical Practice Guidelines for the Diagnosis and Treatment of Postmenopausal Osteoporosis (AACE/ACE) (26)	Adult	BTMs were recommended for initial evaluation and follow-up of patients with osteoporosis and as a target for treatment.
Clinician’s Guide to Prevention and Treatment of Osteoporosis (NOF) (42)	Adult	BTMs can help with risk assessment and serve as an additional monitoring for treatment.
Japanese 2011 guidelines for prevention and treatment of osteoporosis–executive summary (JOS) (43)	Adult	(1) Educate patients who have suboptimal understanding of the need for treatment; (2) In patients who are scheduled to receive pharmacotherapy; (3) When a physician aims to select an appropriate treatment for osteoporosis, as well as to evaluate the response to treatment.
Clinical practice guidelines for the prevention and treatment of osteoporosis in Taiwan: summary (TOA) (44)	Adult	It is recommended to measure BTMs at 3 to 6 months after starting anti-osteoporotic drug therapy.
International Osteoporosis Foundation and European Calcified Tissue Society Working Group. Recommendations for the screening of adherence to oral bisphosphonates (IOF/ECTS) (45)	Adult	BTMs were used to assess treatment adherence.
Consensus Statement on the Use of Bone Turnover Markers For Short-Term Monitoring of Osteoporosis Treatment in the Asia-Pacific Region (46)	Adult	The use of BTM, particularly CTX and P1NP, was endorsed as a short-term monitoring tool.
Guidelines for the Clinical Application of Biochemical Markers of Bone Turnover (China)	Adult	BTMs were used for osteoporotic fracture risk prediction and osteoporosis differential diagnosis
Guidelines for Diagnosis and Treatment of Primary Osteoporosis (China)	Adult	BTMs enable skeletal disorder differential diagnosis, remodeling assessment, fracture prediction, adherence monitoring, and efficacy evaluation (non-diagnostic for osteoporosis).

### 5.1 Bone metabolic diseases

#### 5.1.1 Primary osteoporosis

Osteoporosis was previously recognized as a disease of old age, but is now of great concern in children and adolescents. The major determinant of lifetime risk for osteoporosis is the magnitude of peak bone mass attained in early adulthood, suggesting that bone health in childhood has a significant impact on lifetime risk for osteoporosis and osteoporotic fractures (35, 47). Osteoporosis in children is usually categorized into hereditary primary osteoporosis and secondary osteoporosis caused by systemic disease or drug use. The most common form of primary childhood osteoporosis is impaired osteogenesis, which is primarily associated with mutations in genes involved in the synthesis or post-transcriptional modification of type I collagen (48, 49). The International Society for Clinical Densitometry (ISCD) diagnostic criteria (2013 revision) mandate: (1) ≥1 vertebral compression fracture(s) without underlying focal pathology or high-impact trauma, or (2) low bone mineral density (BMD; Z-score <−2.0) plus significant fracture history (≥2 long bone fractures before age 10 or ≥3 before age 18) (48, 49).

Although not included in osteoporosis diagnostic criteria, BTMs are clinically essential for monitoring treatment response. BTMs are pivotal for monitoring antiresorptive therapy response, with bisphosphonate treatment typically reducing β-CTX by 50-80% within 2 months and PINP by 40-60% within 6 months (14). Multiple international guidelines recognize bone turnover markers (BTMs) as tools for initial osteoporosis assessment and treatment monitoring (**Table 1**). Their superior clinical responsiveness supports therapeutic decision-making. Consequently, the International Osteoporosis Foundation (IOF) and European Calcified Tissue Society (ECTS) recommend BTMs to detect suboptimal treatment adherence (45). International consensus further endorses BTM evaluation of bone remodeling status before initiating anti-osteoporosis therapy, facilitating early assessment of treatment adherence and responsiveness within months of commencement, thereby guiding ongoing therapeutic decisions (46). Specifically, IOF/ECTS advise baseline PINP or β-CTX measurement prior to oral bisphosphonate initiation, with reassessment at 3 months verifying reductions exceeding the least significant change (LSC) (50). Clinically, Denosumab is often used to treat osteoporosis, with transition to bisphosphonate therapy at the end of denosumab treatment. Within days of denosumab treatment, CTX decreases to near undetectable levels and PINP reaches its lowest value within 3–6 months. Without timely transition to bisphosphonate therapy after the final denosumab injection, BTMs rise rapidly, which may be associated with rebound compression fractures. The Endocrine Society therefore recommends the use of BTMs to guide the transition to denosumab therapy. And there are data from studies suggesting that patients who receive PINP monitoring are more likely to start oral bisphosphonate therapy after ending denosumab therapy (14, 51). However, additional studies are needed to validate the clinical usefulness of BTMs, establish standardized reference ranges, and determine the optimal timing and frequency of monitoring.

#### 5.1.2 Rickets

Rickets, a metabolic bone disorder predominantly affecting children and adolescents, arises from vitamin D deficiency or aberrant vitamin D metabolism. Insufficient vitamin D impairs intestinal calcium and phosphate absorption, leading to hypocalcemia and hypophosphatemia that disrupt normal bone mineralization45. In hypophosphatemic rickets, bone formation markers such as OC and ALP may be elevated showing an increase in bone formation stimulation, while a decrease in ALP activity is a good indicator of treatment efficacy. Conversely, bone resorption markers such as NTX may be elevated, reflecting accelerated bone resorption and the extent of bone destruction (52, 53). These dynamic BTM profiles enable quantitative assessment of disease severity and treatment efficacy in rickets management.

#### 5.1.3 Primary hyperparathyroidism

Primary hyperparathyroidism (PHPT) characterized by autonomous overproduction of parathyroid hormone (PTH) from one or more parathyroid glands. Studies have shown that serum markers of bone formation, such as BALP, are often elevated in patients with PHPT, whereas bone resorption markers may not be significantly increased. Early biochemical intervention is critical to mitigate progressive bone loss and osteoporosis risk. Serial BTM monitoring, combined with DXA and BMD assessment, facilitates personalized management to prevent fragility fractures (54, 55). Parathyroidectomy (PTX) normalizes BTMs, with β-CTX restoration to physiological levels indicating successful resolution of PTH-driven bone resorption (55).

PHPT associated with multiple parathyroid gland hyperplasia and/or adenomas (syndromic type) is a multiple endocrine tumor type 1 (MEN 1), the most common endocrinopathy, which is an inherited disorder. Elevated PTH begins in late childhood, adolescence, or early adulthood and may negatively affect the normal acquisition of peak bone mass. It was found that in untreated patients with MEN1-associated PHPT, serum bone alkaline phosphatase (BALP) levels were significantly elevated, showing higher osteoblastic activity. In contrast, in patients with sporadic PHPT, BALP levels were within the normal range. In addition, it was observed that serum PTH, calcium ion, total calcium and BALP levels were significantly reduced in patients with MEN1-associated PHPT and sporadic PHPT after PTX. This suggests that PTX restores normal levels of serum BTMs and reduces bone resorption activity (56). Thus, integrative BTM and BMD evaluation optimizes therapeutic decision-making in PHPT management.

#### 5.1.4 Juvenile idiopathic arthritis

Juvenile idiopathic arthritis (JIA) is a disease associated with imbalances in bone metabolism. Studies demonstrate that BTMs correlate with disease activity and therapeutic response in JIA, BALP levels reflect inflammatory osteoblast activation (57), while increased β-CTX concentrations are associated with adiposity and hyperleptinemia (58, 59). Concomitant reductions in BMD further underscore the elevated osteoporosis and fracture risk in this population (57).

Current data suggest that JIA-associated bone loss arises from a resorption-formation imbalance, driven by chronic inflammation and metabolic disturbances. While BTM profiling provides actionable insights into skeletal health and treatment efficacy, the precise mechanisms linking JIA pathophysiology to bone remodeling remain incompletely elucidated. Targeted interventions to preserve bone mass in JIA, particularly those addressing inflammatory and leptin-mediated pathways, require further translational and clinical validation.

### 5.2 Endocrine diseases

#### 5.2.1 Obesity

Pediatric obesity is associated with suppressed bone turnover, particularly in females, as evidenced by reduced OC, NTX, and OC/NTX ratios. This hypometabolic state may arise from dual mechanisms: (1) impaired osteoblast differentiation or activity due to adipocyte-dominated marrow microenvironmental remodeling; and (2) chronic low-grade inflammation with elevated TNF-α, IL-6 inhibiting Wnt/β-catenin signaling pathways critical for osteogenesis (36). The adipokine imbalance characteristic of obesity, notably hyperleptinemia, further exacerbates skeletal metabolism dysregulation through central nervous system-mediated suppression of bone formation (60).Although these mechanisms collectively suggest reduced bone strength and compromised peak bone mass accrual in obese adolescents, causal relationships remain contentious. Prospective cohort studies are needed to clarify whether obesity-induced bone turnover suppression independently predicts long-term fracture risk or merely associates with established osteoporosis risk factors.

#### 5.2.2 Diabetes

Type 1 diabetes mellitus (T1DM) exerts deleterious effects on pediatric bone health through multifactorial pathways. Chronic hyperglycemia disrupts calcium-vitamin D homeostasis, induces advanced glycation end-product (AGE) accumulation in bone collagen, promotes marrow adiposity via osteoblast-adipocyte transdifferentiation, and exacerbates oxidative stress—collectively impairing osteoblastogenesis while enhancing osteoclastic activity (61). Evidence indicates that T1DM children exhibit suppressed bone remodeling, characterized by reduced bone formation markers (e.g., PINP) and elevated resorption markers (e.g., β-CTX), predisposing them to low BMD and fragility fractures (61, 62).

While optimized glycemic control may attenuate these metabolic perturbations, the precise molecular mechanisms underlying diabetic osteopathy, particularly AGE-RAGE axis activation and Wnt/β-catenin pathway inhibition, require further elucidation. Targeted therapeutic strategies combining glycemia management with bone anabolic agents represent a promising avenue for mitigating skeletal complications in pediatric T1DM.

### 5.3 Tumors

Acute lymphoblastic leukemia (ALL), the most prevalent pediatric malignancy, has achieved 5-year survival rates exceeding 90% through modern therapeutic protocols. However, skeletal changes observed at the time of diagnosis and during treatment negatively impact the skeletal health of patients, including osteolysis, sclerosis, and osteoporosis. The occurrence of these skeletal changes is largely attributed to the disease itself as well as the effects of intensive treatment regimens (e.g., methotrexate and glucocorticoids) on the skeleton (63). It has been found that pediatric ALL survivors continue to face decreased bone density after treatment, but there is a lack of clarity about the timing of the onset of bone loss, recovery, and long-term prognosis. The causes of decreased bone density may be multifactorial, including the disease itself, concurrent infections, malnutrition, decreased physical activity and abnormal vitamin D metabolism, as well as the therapeutic drugs and radiation therapy used (10, 63). Biochemical evidence demonstrates suppressed bone turnover during treatment, characterized by decreased ALP, PICP, and elevated β-CTX indicative of impaired formation and enhanced resorption (64). These alterations underscore the need for targeted skeletal surveillance in ALL survivorship care.

Bone tumors are characterized by pathological disruption of bone remodeling homeostasis, manifesting as imbalanced osteogenic-osteoclastic coupling. In osteosarcoma, BALP levels are consistently elevated compared to benign bone lesions, with meta-analyses demonstrating 3- to 5-fold higher concentrations (65). BALP quantification exhibits particular diagnostic value in radiologically equivocal cases, achieving 82% sensitivity and 91% specificity for differentiating osteosarcoma from benign tumors and osseous metastases in multicenter studies (66). Current consensus guidelines therefore recommend BALP as an adjunctive diagnostic tool rather than a standalone biomarker, requiring integration with imaging and histopathological findings for definitive diagnosis (65).

## 6 Discussion

Although BTMs aid in assessing pediatric bone health, predicting bone loss, and monitoring therapeutic efficacy, their clinical utility is limited by inherent biological variability and methodological differences, necessitating careful interpretation (67). Physiological variability arises from modifiable and non-modifiable factors. Modifiable factors include age, sex, ethnicity, and medication use, underscoring the significant clinical value of establishing pediatric-specific BTM reference intervals (RIs) (68). However, developing robust RIs requires large pediatric cohorts and addresses complex ethical considerations. Per CLSI EP28-A3 guidelines, establishing pediatric RIs mandates stratification by sex and narrow age intervals (e.g., 1–2 years), with each subgroup requiring more than 120 reference individuals to ensure statistical validity and clinical relevance. Furthermore, RIs require periodic reassessment to account for evolving laboratory methodologies (3, 35). Among non-modifiable factors, circadian rhythm exerts the strongest influence, followed by exercise intensity, seasonal variation, and dietary status (37, 67). Consequently, standardized morning fasting blood collection is recommended to enhance measurement consistency (69).

In terms of methodological differences, the clinical application of BTMs faces challenges in terms of standardization and measurement accuracy (70). Currently, the commonly used detection methods for BTMs are enzyme-linked immunosorbent assay (ELISA) and chemiluminescence immunoassay (CLIA) (6, 71). Different manufacturers use their own testing procedures and primary calibrators to assign values to their company’s products, and different laboratories use different measurement methods and units, making it difficult to compare and interpret results. **Table 2** summarizes the pediatric reference ranges in different manufacturers. To address the issues of traceability and standardization of bone turnover marker measurements, it is possible to develop a reference measurement system and produce standard materials. By standardizing BTM measurements, researchers can ensure that the obtained results reflect the true bone turnover status, enabling more accurate diagnosis and monitoring of treatment (72–75). In this regard, the International Osteoporosis Foundation (IOF) and the International Federation of Clinical Chemistry and Laboratory Medicine (IFCC) have convened the IOF-IFCC Bone Marker Standards Working Group for further research. Through developing commutable international reference materials, conducting methodological comparability studies, and standardizing detection protocols, our consortium has markedly improved the reliability and clinical utility of BTMs (76, 77). Consequently, significant age- and sex-dependent variations in BTMs among pediatric and adolescent populations will gain greater clinical relevance through future advances in reference interval establishment and methodological standardization. This progress will enhance BTM utility for growth monitoring, disease diagnosis, and therapeutic evaluation in these cohorts.

**Table 2:** Sudies providing the reference intervals (RIs) of BTMs in different race, gender, ages and manufacturers.

BTMs	Race	Gender	Age	RI	Manufacturers	Detection methods
PINP(ng/ml)	Asian	Female	≤1y	304.8-1998.4	Roche	Electrochemiluminescence
			2–8y	184.7-889.9		
			9–11y	301.6-1177.0		
			12–18y	46.3-495.0		
			Post-menopause	20.00–76.50	Snibe	Chemiluminescence
			Pre- menopause	15.00–59.00		
			Post-menopause	15.98–75.21	UUDIAG	Electrochemiluminescence
			Pre- menopause	14.56–59.62		
			Post-menopause	16.12–73.17	Hotgen	Chemiluminescence
			Pre- menopause	15.25–58.83		
		Male	≤1y	777.7-1321.4	Roche	Electrochemiluminescence
			2–9y	129.0-879.0		
			10–12y	148.7-1322.2		
			13–18y	55.9-679.9		
			n/a	22.59-75.17	UUDIAG	Electrochemiluminescence
	Caucasian	Female	6–10y	411-1022	Roche	Electrochemiluminescence
			>10–11y	0-1451		
			>11–14y	109- 1346		
			>14–15y	38- 510		
			>15y	49- 277		
			(30–89y)Post- menopause	16.27–73.87		
			(30–89y) Pre- menopause	15.13–58.59		
			8-9y	415–1210	IDS	ELISA
			10-11y	352–1513		
			12-13y	387–1439		
			14-15y	65–726		
			16-17y	55–325		
PINP(ng/ml)	Caucasian	Male	6–11y	407- 1079	Roche	Electrochemiluminescence
			>11–14y	339-1399		
			>14–15y	0-1217		
			>15y	61- 718		
			8-9y	381–1138	IDS	ELISA
			10-11y	298–1314		
			12-13y	168–1858		
			14-15y	219–1931		
			16-17y	166–2623	IDS	ELISA
		All	Adults	27.7 – 127.6	IDS	ELISA
OC(ng/ml)	Asian	Female	< & =9y	22.7-90.3	Roche	Electrochemiluminescence
			10–12y	23.7-141.8		
			13–18y	10.9-71.2		
			Post-menopause	14-47	UUDIAG	Electrochemiluminescence
			(>20y) Pre- menopause	10-45		
			Post-menopause	9-47	Snibe	Chemiluminescence
			Pre- menopause	10-38		
		Male	< & =10y	21.7-93.8	Roche	Electrochemiluminescence
			11–14y	31.1-173.7		
			15–18y	68.8-11.3		
			18-29y	22-69	UUDIAG	Electrochemiluminescence
			>29-50y	15-41		
			>50-70y	15-46		
			18-30y	20-59	Snibe	Chemiluminescence
			>30-70y	8-41		
	Caucasian	Female	6-10y	61.4- 136.2	Roche	Electrochemiluminescence
			>10-14y	24.1- 232.1		
			>14-15y	17.8- 119.6		
			>15y	21.1- 76.7		
			Post- menopause	15-46		
			(>20y) Pre- menopause	11–43		
		Male	6–9y	56.5- 152.1		
			>9–15y	48.2-226.4		
			>15y	22.5-151.3		
			18-29y	24-70		
			>29-50y	14-42		
			>50-70y	14-46		
BALP(U/L)	Caucasian	Female	6–11y	23.5- 151.1	Roche	Electrochemiluminescence
			>11–14y	20.8- 172.3		
			>14y–15y	12.6- 105.8		
BALP(U/L)	Caucasian	Female	>15y	8.1-43.9	Roche	Electrochemiluminescence
			< & =1y	117-152	Quidel	ELISA
			2y	113-148		
			3y	129-147		
			4y	109-147		
			5y	108-148		
			6y	106-148		
			7y	104-148		
			8y	101-147	Quidel	ELISA
			9y	96-143		
			10y	90-137		
			11y	81-127		
			12y	71-113		
			13y	59-96		
			14y	46-77		
			15y	35-59		
			16y	26-45		
			17y	20-36		
			18y	17-31		
			19y	15-27		
		Male	6–9y	51.0-164.3	Roche	Electrochemiluminescence
			>9–11y	65.6-138.2		
			>11–15y	45.5- 208.4		
			>15y	13.1-80.0		
			< & =1y	126-155	Quidel	ELISA
			2y	119-148		
			3y	115-143		
			4y	112-141		
			5y	110-139		
			6y	110-140		
			7y	111-142		
			8y	112-146		
			9y	115-152		
			10y	117-158		
			11y	120-165		
			12y	120-169		
			13y	118-171		
			14y	113-168		
			15y	103-157		
			16y	88-137		
BALP(U/L)	Caucasian	Male	17y	69-111	Quidel	ELISA
			18y	47-78		
			19y	25-43		
β-CTX(ng/mL)	Asian	Female	Post-menopause	<1.014	UUDIAG	Electrochemiluminescence
			Pre- menopause	<0.563		
		Male	30-50y	<0.573		
			51-70y	<0.695		
			>70y	<0.835		
	Caucasian	Female	6–10y	0.82- 2.06	Roche	Electrochemiluminescence
			>10–14y	0.49- 2.76		
			>14–15y	0.12- 1.73		
			>15y	0.00- 1.59		
			(30–89y)Post- menopause	<1.008		
			(30–89y) Pre- menopause	<0.573		
			8-9y	1.03-3.20	IDS	ELISA
			10-11y	1.10-3.76		
			12-13y	0.96-3.72		
			14-15y	0.33-2.79		
			16-17y	0.29-1.24		
			Post-menopause	0.142-1.351		
			Pre- menopause	0.112-0.738		
		Male	6–9y	1.05-2.38	Roche	Electrochemiluminescence
			>9–15y	1.00- 2.90		
			>15y	0.50-2.43		
			30-50y	<0.584		
			8-9y	1.08-2.96	IDS	ELISA
			10-11y	1.14–3.36		
			12-13y	1.10–4.06		
			14-15y	1.00–4.39		
			16-17y	1.06–4.90		
			n/a	0.115-0.748		

Studies in perimenopausal women demonstrate a clear association between elevated BTMs and cortical/trabecular bone loss, with particularly strong correlations observed at the spine (37). However, comparable evidence linking BTMs to bone mass changes remains scarce for male and pediatric populations. Future research should establish BTM-bone mass relationships across demographics and identify optimal markers for early diagnosis and treatment monitoring. Complementary approaches, such as Bone Balance Index (BBI) studies, demonstrate utility for predicting bone loss in perimenopausal populations (78). Future efforts should focus on integrating BTMs with other clinical indicators such as bone densitometry to enhance the accuracy and efficiency of diagnosis and monitoring. Combining these different measures can improve the sensitivity and specificity of diagnosis and monitoring, allowing for earlier detection of bone-related disorders and more effective treatment strategies (14). Furthermore, more efforts should integrate combining proteomic BTM profiles with genomic signatures to unravel individualized bone remodeling dynamics. This will be beneficial in bridging the gap between mechanistic insights and clinical translation.
